# Anti-Oxidant and Anti-Cancer Properties of Flaxseed

**DOI:** 10.3390/ijms26031226

**Published:** 2025-01-30

**Authors:** Agnieszka Ewa Stepień, Julia Trojniak, Jacek Tabarkiewicz

**Affiliations:** 1 Institute of Health Sciences, Medical College of Rzeszów University, University of Rzeszow, 35-959 Rzeszów, Poland; agstepien@ur.edu.pl; 2 Student’s Scientific Club of Immunology, Institute of Medical Sciences, Medical College of Rzeszów University, University of Rzeszow, 35-959 Rzeszów, Poland; jt117576@stud.ur.edu.pl; 3 Department of Human Immunology, Institute of Medical Sciences, Medical College of Rzeszów University, University of Rzeszow, 35-959 Rzeszów, Poland

**Keywords:** flaxseed, linseed, common flax, superfoods, *Linum usitatissimum*

## Abstract

Bioactive molecules present in plant products determine their very valuable health-promoting properties. Among the plants, due to these properties, particular attention is paid to the seeds of common flax (*Linum usitatissimum* L.), which have been used for over 6000 years and are known for their benefits. A review of 117 scientific articles indexed in PubMed/MEDLINE, ScienceDirect, and Wiley Online Library, published between 1997 and 2024, was conducted. These seeds are characterized by a high content of valuable nutrients, such as essential omega-3 fatty acids, including α-linolenic acid (ALA), lignans, isoflavones, phytoestrogens, flavonoids, vitamins, and minerals that influence the digestive system function and have anti-cancer properties. The presence of these bioactive compounds in flaxseeds provide anti-cancer properties.

## 1. Introduction

**Figure 1:**
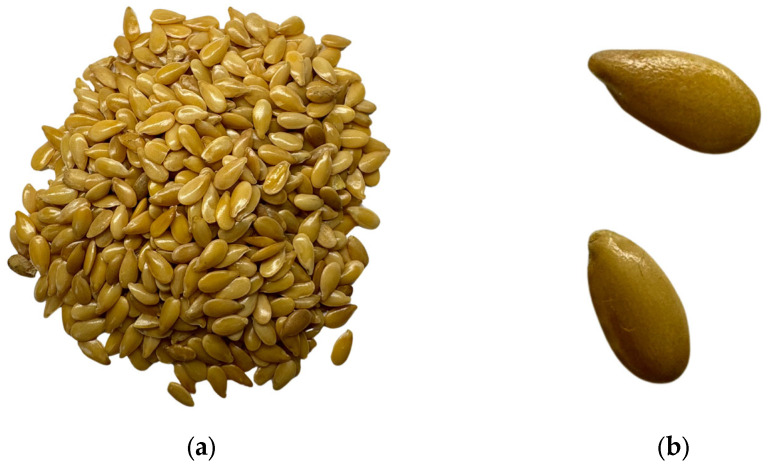
Gold flaxseed (linseed) from *Linum usitatissimum* L. (photographs by J. Trojniak). (**a**) Gold flaxseed (linseed) from *Linum usitatissimum* L. – low magnification, (**b**) Gold flaxseed (linseed) from *Linum usitatissimum* L. – higher magnification.

Common flax (*Linum usitatissimum* L.), belonging to the flax family *(Linaceae)*, is one of the oldest cultivated plants in the world. It is estimated that cultivation in the Middle East dates back to around 9000 BC [1]. Flax is an annual, blue herb that grows in temperate climates, including Europe, Asia, and North Africa [2,3]. Each part of the linseed plant can be used for a range of purposes, including industrial and culinary uses, as well as animal feed [4]. Flax can be cultivated in two forms, depending on the climatic conditions: oily (*Linum usitatissimum* L. var. *brevimulticaulis Vav.*) and fibrous (*Linum usitatissimum* L. var. *elongatum Vav.*) [2,5]. Furthermore, flax comes in two varieties: brown and golden. Golden flax thrives in very cold climates, while brown flax thrives in warmer, more humid climates [6]. Common flax produces small flat and oval seeds with pointed tips that range from golden yellow to reddish brown in color [3,7]—Figure 1. The seed coat of flaxseed consists of a true husk (also known as the kernel), a thin endosperm, and two embryos [8]. Flaxseed has a crunchy texture and a nutty, slightly spicy taste [3,8].

Flaxseed (linseed) is a traditional ingredient in the human diet. It is widely known that linseeds have historically been used as a source of fiber and oil. Still, their exceptional nutritional properties have gained popularity as an important dietary ingredient. More people are becoming aware of flaxseeds’ health-promoting benefits after being used mostly for industrial purposes for decades. A number of the nutrients and bioactive compounds found in linseed benefit human health. Flax components with abundant health-promoting properties include three types of phenolic compounds, phenolic acids, flavonoids and lignans, omega-3 fatty acids, dietary fiber, vitamins, and minerals, which is why they are classified as "superfoods" [3,9]. It is worth noting that flaxseed fiber can support the digestive system’s health by regulating intestinal peristalsis and providing anti-inflammatory effects [10,11].

**Figure 2:**
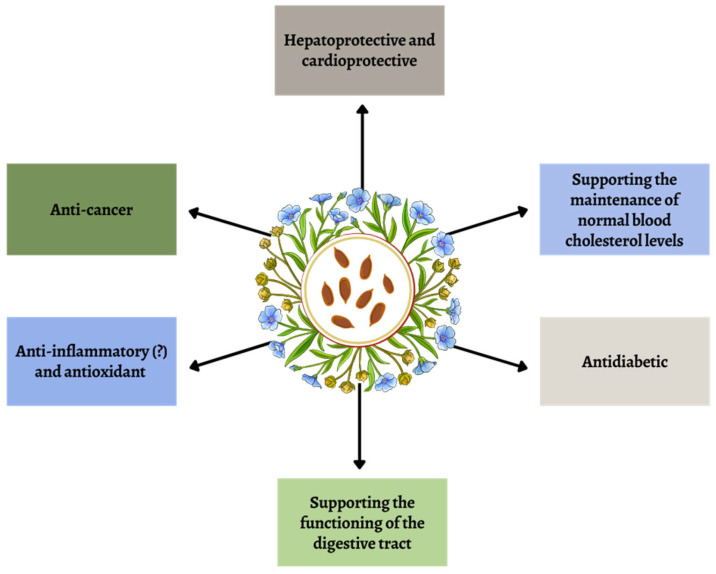
Selected properties of flaxseed (linseed).

Several studies suggest that linseed may be beneficial for heart health due to its antihypertensive, anti-atherosclerotic, and cholesterol-lowering properties [12,13,14]. Among its benefits are also the reduction of lipid levels in the blood, anti-diabetic properties, hepatoprotective properties, and the prevention of many cardiovascular diseases (CVDs) and cancers [15,16,17,18]—Figure 2. Additionally, some reports mention antipyretic action, analgesic effects, reduced symptoms, and improved general functioning, especially in osteoarthritis of the knee joint [19,20]. It is also worth mentioning that flaxseed may contribute to the reduction of inflammatory markers such as CRP and TNF-α [4].

There are documented reports of the positive effects of linseed on human health, including tolerance of linseed by people with chronic or genetic diseases, such as cystic fibrosis [21]. However, special caution should be taken in some populations [21]. Because of its estrogenic effect, women and men who are trying to conceive, and especially pregnant women, should exercise special caution when taking flaxseed [22]. Additionally, α-linolenic acid (ALA) present in linseed is associated with improved brain function and is important for maintaining a healthy nervous system [23].

The benefits of flaxseed have also been established in recent studies, including relief from perimenopausal symptoms [24]. Its alpha-linolenic acid (ALA) content may protect against neurological damage and also improve cognitive function in older people [25]. Additionally, flaxseed may reduce inflammatory biomarkers in cardiovascular patients, offering further benefits to heart health beyond its known cholesterol- and blood pressure-lowering effects [22].

Despite the numerous health and nutritional benefits of flaxseed, it has also been described to contain high levels of toxic compounds that can affect the bioavailability of nutrients and/or further worsen health problems [26]. When comparing the nutrients and antinutrients of flaxseed, it can be concluded that the nutrients significantly predominate, and the level of antinutrients can be significantly minimized by using appropriate methods of flaxseed processing, such as autoclaving or microwave baking [27,28].

The choice to focus on the anti-oxidant and anti-cancer properties stems from their crucial significance in the context of research on the prevention of chronic diseases, such as cancer. Studies indicate that these properties have wide-ranging applications in public health protection, which justifies their detailed analysis in our work. The oxidative stress associated with inflammation, especially chronic, is one of the main factors of carcinogenesis; what causes those anti-oxidative activities of natural compounds are usually associated with anti-cancer properties. We chose to summarize the benefits of common flax as another valuable plant after discussing the benefits of *Sambucus Nigra* and black garlic, two other plants that promote health [29,30]. Our study concentrates on compiling scientific reports on the anti-cancer, anti-inflammatory, and anti-oxidant properties, providing a comprehensive summary of the published literature and highlighting gaps, including those related to safety and long-term adverse effects, which warrant further investigation.

## 2. Methodology and Selection Criteria

Our article presents a comprehensive review of literature published between 1997 and 2024, focusing on the anti-oxidant and anti-cancer properties of compounds found in flaxseed. The analysis was conducted using the following databases: MEDLINE/PubMed, Wiley Online Library, and ScienceDirect. Our work primarily focused on original research articles, as well as the collection and synthesis of previous review articles and meta-analyses. The search criteria included keywords such as “flaxseed”, “*Linum usitatissimum*”, “antioxidant”, and “anti-cancer”, which were combined in various configurations. A search in PubMed using the terms “flaxseed” or “*Linum usitatissimum*” yielded a total of 17,420 results. After applying relevant filters, this number was reduced to 102 articles. The same search strategy was employed in the Wiley Online Library, yielding 118 results, and in ScienceDirect, yielding 23 results.

From the initial pool of 243 articles published between 1997 and 2024, 62 were excluded following title and abstract screening. A more detailed analysis was performed on the remaining 181 articles, evaluating their methodology and results. Further analysis in terms of methodology and results was carried out by reading the full texts of the 181 remaining articles. Finally, after selecting articles whose subject matter was consistent with our review, we analyzed 117 scientific papers.

The exclusion criteria included a lack of detailed information on anti-oxidant or anti-cancer properties, unclear research methods, or data that did not meet scientific reliability standards. Only articles that comprehensively documented the anti-oxidant and anti-cancer effects of flaxseed were included in the final analysis.

## 3. Active Ingredients in Flaxseed

The use of phytochemicals to prevent neoplasms is becoming increasingly popular. This inhibiting effect of phytochemicals on cancerogenesis is frequently associated with anti-oxidative properties. Compared to synthetic anti-oxidants, which often come with undesirable side effects, naturally occurring anti-oxidants are considered a safe alternative [31].

**Figure 3:**
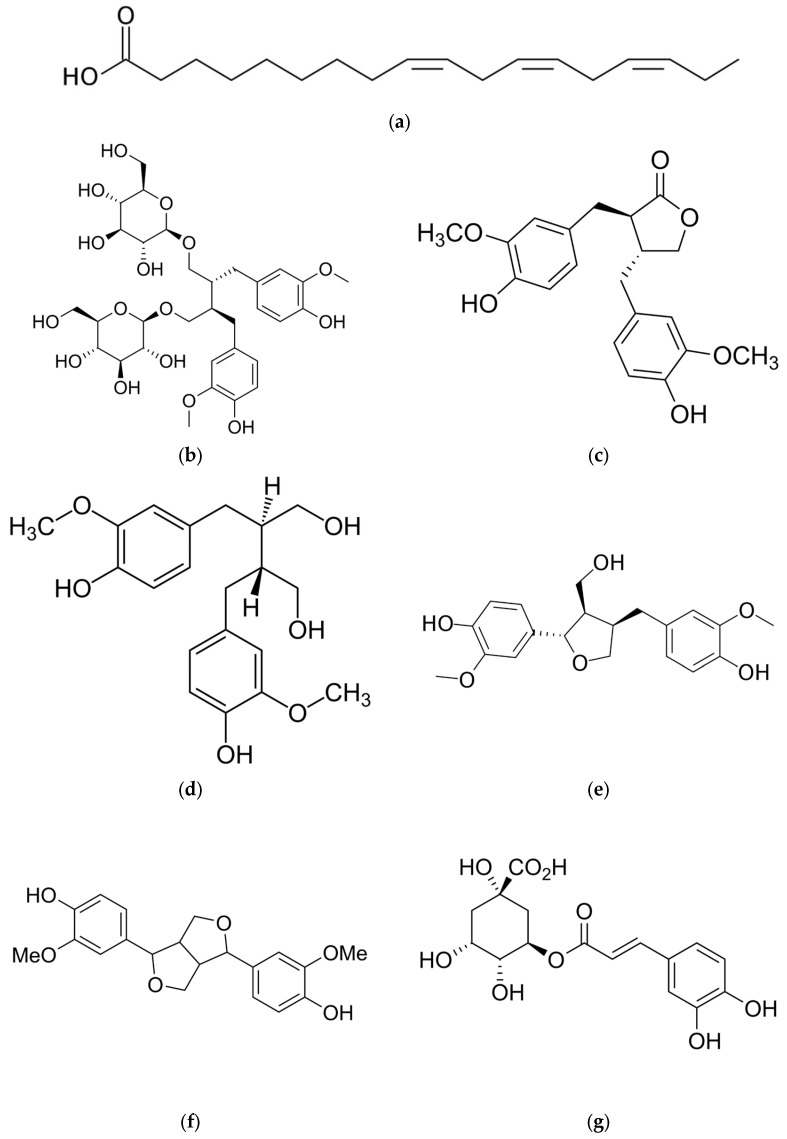
Chemical structures of several of the most important active compounds found in flaxseed from common flax *(Linum usitatissimum* L.): (**a**) α-linolenic acid (ALA); (**b**) secoisolariciresinol diglucoside (SDG); (**c**) matairesinol (MAT); (**d**) secoisolariciresinol; (**e**) laricinesinol; (**f**) pinoresinol; (**g**) chlorogenic acid (CGA).

Flaxseed (*Linum usitatissimum* L.) is a highly nutritious seed containing numerous active ingredients. There is a wide variation in chemical composition among varieties and growing conditions. Flaxseed is naturally enriched with α-linolenic acids (ALAs), short-chain polyunsaturated fatty acids (SC-PUFAs), lignans, and dietary fiber and is a cluster of anti-oxidative and anti-cancer agents and other bioactive compounds [3]—Table 1, Figure 3a–g.

**Table 1:** Active compounds of flaxseed (linseed) from common flax (*Linum usitatissimum* L.).

Component		Contents		Unit	References
humidity		7.13; 5.39–6.04		[%]	[32,33,34]
nitrogen		4.01			
fat		41; 34/08–40/74			
protein		20; 18.9–27.0			
ash		3.4; 3.54–4.49			
digestive fiber total		28			
crude fiber		7.6–11.8			
soluble fibers		4.3–8.6; 9		[g/100 g flaxseed]	
insoluble fibers		12.8–17.1; 20			
fatty acids	α-linolenic acid (ALA)	22.8		[g/100 g flaxseed]	[32]
		42/97–61/06 58.59 ± 0.43; 53.6–57.1		%	[33,35,36]
	acid linoleic	5.9;		[g/100 g flaxseed]	[32]
		9.18–15.88; 15.50 ± 0.21; 14.0–14.4		%	[33,35,36]
	acid oleic	7.3; 16.33–22.56		[g/100 g flaxseed]	[32]
		17.27–20.50; 16.67 ± 0.23; 18.5–21.0		%	[35,36]
	acid stearic	1.3		[g/100 g flaxseed]	[32]
		3.65–5.96; 3.66 ± 0.08		%	[35,36]
	acid palmitic	1.2; 2.1; 4.58–6.42;		[g/100 g flaxseed]	[8,32,36]
		4.58–6.42; 5.44 ± 0.11		[%]	[35,36]
	acid glutamic	19.6		[g/100 g flaxseed]	[32]
	acid aspartic	9.3		[g/100 g flaxseed]	[32]
	saturated (SAFA/SFA)	8.42–12.90; 9.18 ± 0.21; 10.0; 9.23-24.01		[%]	[33,35,36,37]
	total monounsaturated (MUFA)	16.37–23.00; 16.69 ± 0.24; 16.4–23.5; 18.20–23.53; 22.5			
	complete unsaturated (PUFA)	52.15–76.94; 74.09 ± 0.64; 57.8–74.3			
acids phenolic and lignans		Non-defatted extracts	Defatted extracts	[mg/100 g]	[32,38,39]
	p-hydroxybenzoic acid	1719	6454		
	chlorogenic acid (CGA)	720–750	1435		
	ferulic acid	109–161	313		
	coumaric acid	87	130		
	gallic acid	28–29	17		
	vanilla acid	22	42		
	sinapic acid	18	27		
	protocatechuic acid	7	7		
	caffeic acid	4	15		
	diphyllin	4.2	n.a. ^1^		
	secoisolariciresinol diglucoside (SDG)	1300	n.a.		
	secoisolariciresinol	156,165	n.a.		
	lariciresinol	1.7	n.a.		
	matairesinol (MAT)	3.1	n.a.		
	pinoresinol	0.8	n.a.		
Flavonoids total		35–70		mg/100 g of flaxseed linen	[32]
minerals	calcium	170–236		[mg/100 g flaxseed]	[3,32]
	magnesium	431			
	phosphorus	370–622			
	potassium	750–831			
	sodium	27			
	zinc	4			
	iron	2.7–5			
	manganese	3			
Vitamins and dyes	α-Tocopherol	6.26		(µg/g)	[40,41,42]
	β-Tocopherol	1/07			
	γ-Tocopherol	302.0			
	δ-Tocopherol	2.26			
	β-Carotenoid	0.52			
	Xanthophyll	27.1			
	vitamin C	0.5		g/100 g of flaxseed linen	[32]
	vitamin B1	0.5			
	vitamin B2	0.2			
	vitamin B3	1.2			
	vitamin B6	0.6			
	pantothenic acid	0.6			

^1^ n.a.—not available.

One of the key active ingredients found in linseed are omega-3 fatty acids, including monounsaturated α-linolenic acid (ALA). ALA accounts for approximately 55% of the total fatty acid content in linseed seeds [43]. The body cannot produce this fatty acid on its own and needs to obtain it from external sources [44]. Several studies have shown that omega-3 fatty acids are especially helpful for cardiovascular health due to their impact on blood cholesterol levels and their ability to reduce cardiovascular mortality risk [45,46,47]. Furthermore, they regulate metabolic processes and have anti-cancer properties [45]. According to studies, flaxseed consumption has been found to reduce triglycerides as well as improve the blood lipid profile in people with metabolic disorders [22]. As well as being high in protein, linseed also contains high levels of linoleic acid, which contributes to proper cholesterol levels [48,49].

Lignans are diphenolic compounds that are formed from the combining of two coniferous alcohol residues in the cell wall of higher plants [50]. In flaxseeds, the dominant lignan component is diglucoside secoisolaresinol (SDG), which makes up about 95% of the lignan content. The remaining 5% are laryresinol, pinoresinol, and matairesinol [6]. The health benefits of flax lignans are thought to be due to their anti-oxidant activity, primarily as hydroxyl radical scavengers, and their hormone-like activity, in part due to their structural similarity to 17-β-estradiol [50]. The behavior of lignans depends on the biological level of estradiol. With normal estradiol levels, lignans act as estrogen antagonists, but in postmenopausal women when estrogen levels are low, they may act as weak estrogens [47]. A combination of bioactive polyphenols and lignans found in linseed, including the anti-oxidant secoisolariciresinol diglucoside (SDG), as well as its fiber content, benefit the cardiovascular system [51].

Mammalian lignans enterolactone (EL) and enterodiol are produced by the action of bacteria in the colon on SDG. Case-control studies have shown a significant inverse relationship between breast cancer risk and urinary enterolactone levels [52,53].

Researchers have also found that linseed may have an antiviral effect, particularly against HIV. Several studies have shown that lignans found in linseed can inhibit HIV virus replication, giving hope for its use in treatment and prevention [54,55].

The orbitides found in flaxseed and flaxseed rhizome are hydrophobic cyclic peptides that contain eight or nine amino acids without cysteine [56]. More than 20 linseed orbitides have been identified from isolated linseed oil products to date [56].

There is also insoluble fiber in flaxseed, which contributes to the high nutritional value of the seed. This fiber is necessary for the proper functioning of the digestive system by maintaining proper intestinal peristalsis. It also provides a feeling of satiety, which may be especially helpful when losing weight. In some studies, flaxseed fiber has been shown to prevent digestive disorders such as constipation and even to reduce the risk of colon cancer [10].

Besides the active ingredients mentioned above, flaxseed also contains numerous valuable compounds, including vitamins (A, E, B), minerals (magnesium, iron, zinc), and flavonoids—Table 1. All of these nutrients play an important role in strengthening the immune system; improving the skin, hair, and nails; and contributing to the healthy function of the nervous system.

## 4. Anti-Oxidant Properties

A cellular inflammatory response is a complex reaction created by both external and internal factors, and it aims to eliminate the cause of the damage and its negative effects on cell function. In pathological inflammation, phagocytosis-deficient cells and those that cannot phagocytose produce an excess of reactive oxygen species (ROS) and nitrogen species (RNS). These substances spread into the intercellular space, leading to local oxidative stress and tissue damage [57,58]. The main pathway of oxidative damage is hydroxyl-induced lipid peroxidation and hydroxylation of free radicals (OH∙). As a result of lipid peroxidation, membrane fluidity decreases; membrane leakage increases; membrane proteins are damaged; and receptors, enzymes, and ion channels are inactivated [59]. Excessively active or prolonged inflammation may contribute to the development of various chronic illnesses [60].

Linseeds contain a large amount of phenolic compounds. It is well known that these phenolic compounds have anti-cancer as well as anti-oxidant properties. Numerous clinical studies have demonstrated the anti-inflammatory potential of n-3 polyunsaturated fatty acids mediated by the decrease in concentrations of inflammatory mediators, such as prostaglandins E2, leukotriene B4, TNF-α, interleukins, and cytokines [3].

The analysis showed that in overweight people (BMI >30), linseed consumption resulted in a reduction of the levels of TNFα, CRP, and hs-CRP compared to the placebo group. However, no significant differences were observed in other population subgroups. Additionally, in people with a higher BMI, flaxseed slightly reduced the level of IL-6, although this effect was not significant [60]. In rodents, a diet enriched with α-linolenic acid, a component of linseed, reduced oxidative stress and inflammation during myocardial infarction. The linseed component, n-3 PUFA (ALA), provided heart protection. Flaxseeds also contain high levels of functional phenolic compounds that may increase the cardioprotective effect [61]. Additionally, the linoleic acid metabolite 10-hydroxy-cis-12-octadecenoic acid (HYA) has been shown to reduce colonic damage in dextran sodium sulfate (DSS)-induced colitis by modulating inflammatory factors, oxidative state, and colonic microbiota imbalance. It produces the effect by inhibiting the expression of TNF-α and changes in the expression of intestinal epithelial tight junction (TJ) proteins such as occludin, occludin-1, and myosin light-chain kinase [62].

The consumption of flaxseed has been shown to reduce insulin resistance, lipid abnormalities, atherosclerosis, hypertension, and cardiac arrhythmias, possibly by improving anti-inflammatory cytokine levels and insulin sensitivity [63]. The benefits of linseed oil on reducing chronic inflammation and reducing CVD risk have also been indicated in hemodialysis patients, namely, a reduction in CRP and hs-CRP levels and a reduction in sVCAM-1 levels [45,46,47]. Furthermore, linseed extract has been reported to reduce TNF-α levels and alleviate the severity of colitis [47].

By containing compounds such as SDG and ALA, flaxseed affects genes involved in inflammation control in obese animals. As reported by Mann et al., supplementation with flaxseed suppresses mild obesity-related inflammation by suppressing Akt2 protein expression, which reduces the activation of the IKKβ/NF-κB inflammatory pathway and the production of pro-inflammatory cytokines [64].

Flaxseed peptides exhibited anti-oxidant properties by scavenging hydroxyl radical (OH∙) and inhibiting nitric oxide (NO) production in macrophages [65]. Moreover, it was also observed that linseed extract showed anti-oxidant capacity in the test with stable free radical molecules (DPPH). The observed effect explains the potential mechanism of the positive effect of linseed on wound healing. In animal studies, flaxseed products caused fibrous tissue to be formed and collagen to migrate, indicating that they may be a safe and effective product for repairing and regenerating the skin [66,67]. Yu et al. reported that oral administration of flaxseed SDG reduced skin inflammation and down-regulated JAK/STAT6 signaling pathways to reduce Th2 immune responses in mice with atopic dermatitis (AD) [68].

In mice exposed to asbestos, which is a risk factor for cancer, including pleural mesothelioma, synthetic diglucoside linseed secaisolaricoresinol (SGD) reduced peritonitis and white blood cell influx. A study showed that SGD inhibited both granulocyte and macrophage infiltration into the peritoneum [69].

Its anti-inflammatory properties are also attributed to its ability to suppress the release of inflammatory mediators, such as prostaglandins E2, histamine, leukotrienes B4, and bradykinin. Furthermore, flaxseed oil inhibits the inflammatory process induced by arachidonic acid, affecting cyclooxygenase and lipoxygenase, which are involved in arachidonic acid metabolism [20].

Different results are reported by Aguilar et al. in their experiment; linseed supplementation had no significant effect on pro-inflammatory biomarkers, including TNF-α; IL-1β and IL-6; and anti-inflammatory biomarkers, such as IL-10 in overweight perimenopause-aged women [70]. Similar conclusions were reported by Shareghfarid et al., in which the use of linseed had no significant effect on body weight and inflammatory markers in overweight and prediabetic adults [71]. Another study found that in normal-weight people following a healthy diet, linseed oil supplementation also did not affect inflammatory markers and lipid profiles [72].

Biao Y et al. reported that polysaccharides fraction isolated from flaxseed hull and composed of eight kinds of monosaccharides—mannose, rhamnose, galactose, glucose, galactose, xylose, arabinose, and fucose—expressed potent anti-oxidant and anti-inflammatory properties, probably attributed to its effects on the NO and MAPK pathway [73]. Another possible mechanism of reducing the release of free radicals and inhibiting macrophages function is modulation from the TLR4/NF-κB/MAPK pathway by flaxseed linusorbs [74]. The findings of Huang X. et al. confirmed the anti-oxidant activity of flaxseed polyphenols [21]. They also analyzed the phenolic acids and flavonoids content in flaxseeds and revealed significant differences in polyphenol quantities and types among different flaxseed varieties, while sinapic acid, ferulic acid, kaempferol, and quercitrin were found to be the most abundant phenolic compounds. The summary of potential molecular mechanisms of anti-oxidant and anti-inflammatory effects of flaxseed are summarized in Table 2.

In the majority of the studies, whole flaxseeds or extracts were used, not isolated and a purified particular compound or combination of compounds; this model reflects dietary supplementation of flaxseeds. On the other hand, the separate compounds responsible for particular molecular mechanisms of anti-oxidant activities are poorly identified, which discourages the further use of a single compound in a higher concentration in supplementation or dietary treatment.

**Table 2:** The effects of flaxseed in anti-oxidant and anti-inflammatory activity and their potential mechanism.

Material Type	Effect	Possible Molecular Mechanism	References
In vitro—RAW264.7 cells	anti-oxidant and anti-inflammatory	impact on the NO and MAPK pathways	[73]
In vitro—RAW264.7 cells	anti-inflammatory	inhibitory effect on TLR4, inhibition of transduction of the NF-κB/MAPK signaling pathway	[74]
Human clinical trial (patients with CF ^1^)	anti-inflammatory, decrease in IsoP and TNFα	ROS sweeping, Nrf2 path activation	[21]
Animal model	anti-inflammatory	stopping ROS and acting against the IKKβ/NF-κB signaling pathway	[64]
Meta-analysis	anti-inflammatory	decrease in TNFα, CRP, and hs-CRP	[60]
In vitro—RAW 264.7 cells	anti-oxidant	regulation of SOD, GSH, and CAT activity; mitigation of the decline in intracellular GSH levels; counteraction of the increase in ROS and MDA levels	[75]

^1^ CF—cystic fibrosis.

## 5. Anti-Cancer Properties

Numerous scientific studies have confirmed that the health-promoting properties of linseed can support human health. Taking into account flaxseed’s health-promoting properties, researchers are testing flaxseeds for anti-cancer properties.

There is evidence that linseed proteins may have anti-cancer properties, suggesting they may be used in cancer treatments in the future [76]. Additionally, linseed is characterized by anti-oxidant properties, which may significantly reduce the severity of cancer-related symptoms due to the association of imbalances in oxidation reactions and anti-oxidant mechanisms [10].

Flaxseed is also rich in lignans, which are important polyphenols in the context of anti-cancer effects. Plant substances such as polyphenols are also generally considered to have anti-inflammatory, antiviral, antibacterial, etc. effects [77]. Lignans contained in linseed also have anti-cancer properties and may be promising chemotherapeutic/chemopreventive substances [39].

The introduction of linseed orbitides into the body has health potential, especially a strong anti-cancer effect [78]. Flaxseed is considered a superfood for its health properties, and regular consumption helps regulate cancer-related genes [79]. Therefore, linseed can be a valuable addition to the diet of people who want to help fight or prevent cancer.

Studies have confirmed the anti-cancer effect of substances contained in linseed, among others, against breast cancer cells, cervical cancer cells, ovarian cancer, colon cancer, leukemia cells, and melanoma cells [80]—Table 3. Studies have also shown that linseed derivatives, for example, oil linseed, inhibit melanoma metastasis and reduce the growth of lung cancer, which confirms their protective effect against cancer [81]. Very interestingly, it also turned out that the growth of non-cancerous cells was not inhibited by treatment with linseed oil, which indicates that linseed and its products preferentially kill malignant cells, without toxicity to normal cells [80].

The lignans enterodiol and enterolactone are believed to be partially responsible for inhibiting the growth of human prostate cancer [3]. Additionally, flaxseed ligands may have non-estrogenic effects, including anti-oxidant activity and inhibition of angiogenesis and cell membrane ATPase [82].

In the study by Sung et al., the anti-cancer effect of linusorb LOB3 ([1–9-Nα C]-linusorb B3), a natural peptide found in linseed, has been demonstrated by inducing apoptosis and inhibiting the motility of cancer cells, especially glioma cells [83]. However, it is worth emphasizing that intravenous infusion of LOB3 may be toxic to humans and may not be appropriate for the treatment of human breast cancer or melanoma. However, the cream formulation of LOB3 may have topical use against melanoma and possibly other types of skin cancers [84].

The anti-proliferative effect of linseed has also been demonstrated in patients with prostate cancer before surgery. A potential mechanism of action may be down-regulation of Ki-67 expression [85].

Linseed and its oil reduce the growth of tumors in the later stages of carcinogenesis, while the mammalian lignan precursor exerts the greatest inhibitory effect on the growth of new tumors [3].

The role of linseed oil in cancer prevention is attributed to its high level of α-linolenic acid. The fatty acid composition of the tumors revealed a higher incorporation of α-linolenic acid, which in turn resulted in the inhibition of tumor cell growth [3]. α-linolenic acid has been shown to exert anti-inflammatory effects and has been shown to have anti-proliferative effects in animal models of premenopausal (high-estrogen) breast cancer [86].

Epidemiological studies indicate that a diet rich in phytoestrogens reduces the risk of various hormone-dependent cancers, heart disease, and osteoporosis [3].

A very interesting relationship was also demonstrated in which linseed oil increased the effectiveness of trastuzumab (TRAS) in reducing the growth of breast tumors (BT-474) with HER2 overexpression at high levels of E2 in the circulation [87].

Flaxseed also led to the suppression of target genes, including potent oncogenes, repressors that target inhibitory cytokine receptors, important growth signaling mediators, proliferative and anti-apoptotic factors, and steroid receptors that are up-regulated in cancer, according to Dikshit et al. [88]. There was also a change in molecular targets involved in inflammation, glucose metabolism, and apoptosis, indicating the anti-cancer and anti-inflammatory effects of linseed in pre-cancerous ovaries [89].

**Table 3:** The effects of flaxseed in anti-cancer activity and their potential mechanism.

Type of Cancer	Cell Line	In Vitro/Animal Model	Active Compounds	Effect	Possible Molecular Mechanism	References
breast cancer HER2(+)	BT-474	animal model – mice	Freshly cold-pressed FSO^1^ containing 9% saturated fat, 18% monounsaturated fat, 14% linoleic acid, and 58% ALA^2^	Reduction of tumor size, increase in tumor cell apoptosis, and reduction of their proliferation	Decreased HER2 signaling through pathways including MAPK and the PI3k–Akt kinase signaling pathway, decreased expression of other growth factor receptors EGFR and IGFIR	[87]
breast cancer ER(−)	MDA-MB-435	animal model – mice	10% freshly ground flaxseed, detailed composition NA^3^	Reduction of tumor growth rate, inhibition of metastases	Reducing the expression of IGF-I and EGFR	[82]
melanoma, breast cancer, cervical cancer	B16-BL6, MCF-7, MDA-MB-231, MDA-MB-468, HeLa	in vitro	Flaxseed oil or a combination of individual fatty acids: ALA^2^, DHA, EPA, linoleic acid, oleic acid, and palmitic acid and the lignans enterodiol and EL^4^	Inhibiting the proliferation of cancer cells and promoting their apoptosis	Promoting apoptosis by activating nuclear fragmentation, increasing the activity of caspase-3 and caspase-8	[80]
ovarian cancer	primary cancer	animal model – chickens *(Gallus domesticus)*	11 grams of flaxseed per day, or 6.2g/kg body weight, detailed composition NA^3^	Reducing the incidence and severity of ovarian cancer	Reduction of COX-2 levels and PGE2 expression	[89]
ovarian cancer	primary cancer	animal model – chickens *(Gallus domesticus)*	11 grams of flaxseed per day, or 6.2g/kg body weight, detailed composition NA^3^	Chemosuppressive effect, inhibition of metastases, slowing down the rate of tumor growth	Anti-oxidant effect	[90]
melanoma, breast cancer	A375, Sk-Br-3, MCF7	in vitro	Linoorbitides: LOB3 (NαC-[Ile-Leu-Val-Pro-Pro-Phe-Phe-Leu-Ile]), LOB2 (NαC-[Met-Leu-Ile-Pro-Pro-Phe-Phe-Val-Ile]), [MetO]-LOB2 (NαC-[MetO-Leu-Ile-Pro-Pro-Phe-Phe-Val-Ile]) and [MetO]-LOB1 (NαC-[Met-Leu-Val-Phe-Pro-Leu-Phe-Ile])	Cytotoxicity towards cancer cells	Cell type specific, probably hydrophobic interaction with the cell membrane, anti-oxidant potential	[84]
glioma, breast cancer	human glioma cell line C6, U251, MDA-MB-231	in vitro	LOB3 ([1–9-NαC]-linusorb B3)	Promoting apoptosis, inhibiting cell motility, preventing metastasis	Inhibition of SRC and STAT3 activation, inhibition of actin polymerization, inhibition of Bcl-2 and Bc1, activation of caspase-9 and caspase-3	[83]
melanoma	B16BL6	animal model – mice (C57BL/6)	Flaxseed containing 2.93 μmol/g of SDG^5^	Reducing the number, cross-sectional area, and volume of metastatic tumors	NA^3^	[91]
colon cancer	primary cancer	animal model – Sprague Dawley rats	SDG^5^-rich extracts (17.57 μg of SDG in 1 mg)	Chemopreventive effect	Inhibition of CDK4, reduction of TNF-α and IL-1β levels	[92]
breast adenocarcinoma	MCF-7 (HTB-22)	in vitro	Flaxseed extract containing hexadecanoic acid methyl ester (9.52%), methyl stearate (9.25%), trans-13-octadecenoic acid methyl ester (22.54%), 9,12-octadecadienoic acid (Z, Z)-methyl ester (19.02%), 9,12,15-octadecatrienoic acid methyl ester (Z, Z, Z)	Inhibiting the growth of cancer cells and inducing apoptosis	Increase in lipid peroxidation; significant increase in p53 gene expression and weak increase in MDM2; increase in levels of cleaved caspase 3, cleaved-PARP, as well as cleaved caspase 7	[93]
acute myeloid leukemia	KG-1 and Monomac-1 acute myeloid leukemia cell lines	in vitro	SDG^5^, END^6^, and EL^4^; EL^4^ was the major compound that showed a dose-dependent and time-dependent anti-proliferative effect	Antiproliferative effect, promoting apoptosis	Up-regulation of Bax and down-regulation of Bcl-2, increase in Bax/Bcl-2 ratio, overexpression of lower cytochrome C molecule, increase in Caspase 9 activation	[94]
prostate adenocarcinoma	primary cancer	animal model – TRAMP and C57BL/6 transgenic mice	Flaxseed, detailed composition NA	Inhibiting the growth and development of cancer cells	Ki-67 level reduction	[95]
breast adenocarcinoma, hepatocellular carcinoma, colon cancer	MCF-7, HepG-2, HCT-116	in vitro	Fs-AuNPs—flaxseed gold nanoparticles	Non-cytotoxic, anti-oxidant effects, induction of apoptosis of cancer cells	NA	[96]
breast cancer	MDA-MB-435	animal model - athymic nude mice (Ncr nu/nu)	10% freshly ground flaxseed and SDG^5^	Inhibition of distant metastases, little effect on locoregional tumor recurrence	Hormone-dependent mechanisms, anti-oxidant, inhibition *inof* signal transduction pathways modulated by IGF-I and EGFR expression	[97]
ovarian adenocarcinoma	BG1FR	animal model – chickens	Whole flaxseed	Reducing prostaglandin production, promoting apoptosis through 2-methoxyestradiol, reducing the aggressiveness and invasiveness of tumors	Increased CYP1A1 expression, increased p38 and ERK 1/2 MAPK activation in the ovary, SMAD 7, decreased 16-hydroxyestradiol levels, increased 2-methoxyestradiol levels	[98]
liver cancer	HepG2	in vitro	Non-oxidized flaxseed orbitides (CLA and CLB) and the oxidized flaxseed orbitides (CLC and CLK)	Inducing apoptosis of cancer cells	Increased protein levels of Bax/Bcl-2, Cyto C, caspase-3, and caspase-8	[78]

^1^ FSO—flaxseed oil, ^2^ ALA—α-linolenic acid, ^3^ NA—not available, ^4^ EL—enterolactone, ^5^ SDG—secoisolariciresinol diglycoside, ^6^ END—Enterodiol.

It is also worth mentioning the study by Asselin et al., which showed for the first time that preventive treatment with linseed, including its components α-linolenic acid (ALA) and diglucoside secoisolaresinol (SDG), significantly alleviated the cardiotoxic side effects of doxorubicin + trastuzumab in a chronic female in vivo model of chemotherapy-induced cardiomyopathy. Flaxseed prevented adverse left-ventricular remodeling; alleviated myofibrillar dysfunction; reduced the degree of inflammation after treatment; and attenuated the degree of cardiac apoptosis, oxidative stress, and mitochondrial dysfunction [99].

There are a number of factors that contribute to the development of cancer, such as oxidative stress, inflammation, and cellular malfunction. Research indicates that flaxseed’s active ingredients, such as lignans and proteins, may have promising properties. In recent years, researchers have identified lignans in linseed as potential chemotherapeutic and chemopreventive agents. These compounds may be used to treat cancer and prevent its occurrence in the future. Cancer cells are inhibited by lignans and undergo apoptosis, a natural death process, as a result of their presence. One of the lignans present in linseed, secoisolariciresinol diglucoside (SDG), has been identified as a strong inhibitor of the 5α-reductase enzyme, which in some cases may contribute to the development of prostate cancer. Additionally, linseed also contains other substances with anti-cancer properties, such as α-linolenic acid and lignin. Also, flaxseeds are rich in fiber, which aids digestion and reduces the risk of cancer, such as colon cancer, among others.

Combining flaxseed with other drugs has also been proven to produce an anti-cancer effect. In mice with transplanted colon cancer, combining linseeds with cyclophosphamide significantly increased the effectiveness of treatment. It appears that linseed could impact cancer treatment by reducing doses of drugs and thereby limiting toxicity.

Another important property of linseed, although questioned in some studies, is its ability to reduce inflammation in the body. It is well know that chronic inflammation can induce cancerogenesis.

The main compound of flaxseed responsible for potential inhibition of inflammation is α-linolenic acid, which is an unsaturated fatty acid. Additionally, it is a precursor to eicosanoids, which are important mediators of inflammatory processes. Some studies have shown that consuming linseed may reduce the concentration of pro-inflammatory cytokines and the activity of factors responsible for the development of inflammation, such as NF-κB. However, other studies report different results, suggesting that the potential effects of linseed and its products are not clinically significant and therefore do not work as anti-inflammatory agents. We suggest that the lack of anti-inflammatory effects of linseed in some studies could have several reasons: First, due to individual differences, flaxseed supplementation may vary according to a person’s genetic makeup, health status, age, diet, and lifestyle [100,101]. Secondly, an insufficient dose of linseed or its components could be the cause. To achieve anti-inflammatory effects, a certain amount of flaxseed may be required, and the doses used by some people may be too low to be effective [102,103]. As a third factor, poor bioavailability may have been responsible [104]. α-linolenic acid (ALA) must be converted in the body to more active forms of omega-3 acids, such as eicosapentaenoic acid (EPA) and docosahexaenoic acid (DHA). Despite this, this process is not equally effective for everyone, especially if omega-3 fatty acids levels are not low [105]. Assimilation and absorption are other possible reasons. Ground flaxseeds (or carefully chewed flaxseeds) can be absorbed by the body more effectively [106]. Otherwise, unground seeds may pass through the digestive system almost unchanged and have no effect. Next, it is worth considering potential interactions with other dietary ingredients. A diet high in other fatty acids, especially omega-6, may increase inflammation in the body, even when consuming flaxseed [107]. Inflammation of high intensity could also contribute to the problem. Compared with the intensity of inflammation in some patients, especially those with chronic inflammation or autoimmune diseases, flaxseed may have a relatively weak anti-inflammatory effect. Our last consideration is lignan metabolism. It has been suggested that the microbiota of the gut may determine the degree of effectiveness of the lignans in flaxseed [108,109].

## 6. Non-Nutritive Ingredients of Flaxseed

Despite its numerous health benefits, flaxseed also contains bioactive compounds that can produce harmful health effects, particularly when overconsumed or improperly processed. Several potentially harmful components present in flaxseed have been highlighted in the scientific literature, including cyanogenic glycosides, proteolytic enzyme inhibitors, and antinutrients such as phytic acid and oxalates [110].

### 6.1. Cyanogenic Glycosides

One of the bioactive compounds present in flaxseed is cyanogenic glycosides, the most prominent being linustatin, neolinustatin, and linamarin [111]. The average content of cyanogenic glycosides in flaxseed is around 100–300 mg hydrogen cyanide (HCN) per kilogram of seeds, which limits the recommended daily intake of flaxseed [112]. Mature seeds contain fewer glycosides compared to unripe seeds. In the presence of plant enzymes or intestinal microflora, these glycosides can be hydrolyzed into toxic hydrogen cyanide (HCN), which is further metabolized into thiocyanates via intestinal β-glycosidase [3,26]. Studies indicate that consuming large quantities of unprocessed flaxseed can lead to hydrogen cyanide poisoning, which may manifest as loss of appetite, lethargy, and neurological disorders [39]. Furthermore, thiocyanates have been reported to inhibit iodine uptake by the thyroid gland, exacerbating iodine deficiency disorders such as goiter and cretinism with long-term exposure [3]. However, it is worth noting that the toxic effects of hydrogen cyanide from flaxseed are relatively rare and primarily occur in cases of excessive consumption or improper meal preparation.

### 6.2. Phytic Acid

Phytic acid is another bioactive component of flaxseed that can negatively affect health. It is well known for its antinutritional properties, as it forms stable complexes with minerals like calcium, iron, magnesium, and zinc, thus hindering their absorption in the small intestine [3].

### 6.3. Proteolytic Enzyme Inhibitors

Flaxseed contains inhibitors of proteolytic enzymes, including trypsin, which can interfere with protein digestion. These inhibitors block the activity of proteolytic enzymes, impeding the breakdown of proteins in the gastrointestinal tract and reducing their bioavailability. Long-term consumption of large amounts of these inhibitors may contribute to protein deficiencies, particularly in individuals whose diet is low in other sources of complete protein [113].

### 6.4. Effects on Hormonal Balance

Flaxseed also contains lignans, which are phytoestrogens, plant compounds that mimic the action of estrogens. While flaxseed lignans have been shown to provide health benefits, such as reducing the risk of hormone-dependent cancers, concerns have been raised regarding their impact on hormonal balance [48]. High lignan intake may lead to hormonal disruptions, especially in individuals with pre-existing hormonal disorders or in children during developmental stages. However, these effects are dose-dependent and vary according to individual sensitivity to phytoestrogens [114].

## 7. Conclusions

Flaxseed (*Linum usitatissimum* L.), one of the oldest cultivated plants, has been used for centuries in traditional medicine. Recent scientific research has increasingly confirmed its anti-cancer properties, particularly, its effectiveness in preventing hormone-related cancers such as breast and prostate cancer, and its ability to inhibit tumor growth. These effects are largely attributed to its rich content of omega-3 fatty acids, lignans, and polyphenols, which help reduce oxidative stress, inflammation, and the risk of chronic diseases.

Flaxseed’s dual role as both a dietary supplement and therapeutic agent makes it a valuable tool in health promotion and disease prevention. However, the anti-inflammatory effects of flaxseed have been reported inconsistently, and the lack of a strong anti-inflammatory response could limit its preventive impact on cancer development. Additionally, flaxseed’s health benefits may take time to manifest, and its effects are best realized when combined with other healthy lifestyle changes, such as a balanced diet and regular physical activity. Despite its many health benefits, there are some concerns about flaxseed due to the presence of antinutritional substances like phytic acid and cyanogenic glycosides, which can be toxic in large amounts. However, proper processing methods can significantly reduce these compounds, enhancing both the nutritional value and safety of flaxseed. Further research is needed to refine consumption guidelines, ensuring maximum benefits while minimizing risks associated with overconsumption or improper use.

## Data Availability

Not applicable.

## Abbreviations

Akt2	ACT Serine/Threonine Kinase 2
ALA	α-linolenic acid
Bc1	complex III or ubiquinol cytochrome C oxidoreductase or cytochrome bc1
Bcl-2	B-cell CLL/lymphoma 2
CDK4	cyclin-dependent kinase 4
CF	cystic fibrosis
CGA	chlorogenic acid
COX-2	cyclooxygenase 2
CRP	C-reactive protein
CVD	cardiovascular disease
Cyclo C	cytochrome C
DHA	docosahexaenoic acid
DSS	dextran sodium sulfate
EGFR	epidermal growth factor receptor
EL	enterolactone
EPA	eicosapentaenoic acid
HER2(+)	HER2-positive breast cancer
Hs-CRP	highly sensitive C-reactive protein
HYA	10-hydroxy-cis-12-octadecenoic acid
IGF-I	insulin like growth factor 1
IGFIR	insulin like growth factor 1 receptor
IKKβ	IκB Kinase β
IL-1β	interleukin 1β
IL-6	interleukin 6
IL-10	interleukin 10
LOB3	[1–9-N α C]-linusorb B3
MAPK	mitogen-activated protein kinases
MAT	matairesinol
NF-κB	the nuclear factor kappa B
PGE2	prostaglandin E2
PI3k-Akt	the phosphatidylinositol 3-kinase (PI3K)/protein kinase B (AKT)
SDG	diglucoside secoisolaresinol
SRC	proto-oncogene tyrosine-protein kinase Sr
STAT3	signal transducers and activators of transcription 3
sVCAM-1	circulating vascular cell adhesion molecule-1
TNF-α	tumor necrosis factor α
TRAS	trastuzumab
